# Isopropanol production from carbon dioxide by *Cupriavidus necator* using a zero-gap cell with culture broth as catholyte

**DOI:** 10.1016/j.isci.2025.113018

**Published:** 2025-06-27

**Authors:** Pierre Schoenmakers, Ramineh Rad, Axel Ihl, Isabell Weickardt, Stéphane Guillouet, Ulf Peter Apfel, Lars Lauterbach

**Affiliations:** 1 RWTH Aachen University, Institute of Applied Microbiology, Worringerweg 1, 52074 Aachen, Germany; 2 Ruhr-University Bochum, Activation of Small Molecules/Technical Electrochemistry, Universitätsstraße 150, 44801 Bochum, Germany; 3 Université de Toulouse, CNRS, INRAE, INSA, Toulouse Biotechnology Institute (TBI), 135 Avenue de Rangueil, 31077 Toulouse, France; 4 Fraunhofer Institute for Molecular Biology and Applied Ecology IME, Forckenbeckstr. 6, 52074 Aachen, Germany

**Keywords:** Chemistry, Microbiology, Chemical Engineering

## Abstract

Microbial CO_2_ fixation is a promising route for sustainable chemical production. The facultative chemolithoautotroph *Cupriavidus necator* can convert CO_2_ into value-added compounds using H_2_ and O_2_. Here, we developed a bioelectrochemical system (BES) for isopropanol production that integrates CO_2_ fixation with *in situ* H_2_ generation via a zero-gap proton exchange membrane electrolyzer. Using the culture broth as catholyte, a pentlandite-based cathode supplied dissolved H_2_ directly to engineered *C. necator*, eliminating the need for pressurized gas. Real-time monitoring of dissolved and off-gas compositions guided system operation, yielding an isopropanol titer of 1.1 g L^−1^. The pentlandite electrode remained stable with negligible leaching. This platform demonstrates a scalable approach for coupling electrolysis and microbial synthesis and can be extended to other H_2_-utilizing microbes.

## Highlights

• PEM-based BES provides safe, *in situ* H_2_ for isopropanol production by engineered *C. necator*
• Pentlandite catalyst supports stable HER in biologically relevant media as catholyte
• Gas analysis reveals how *C. necator* metabolism interacts with supplied H_2_, O_2_, and CO_2_
• Isopropanol titer of 1.1 g L^−1^ achieved from CO_2_ and electricity without organic substrates

## Graphical abstract

**Figure:**
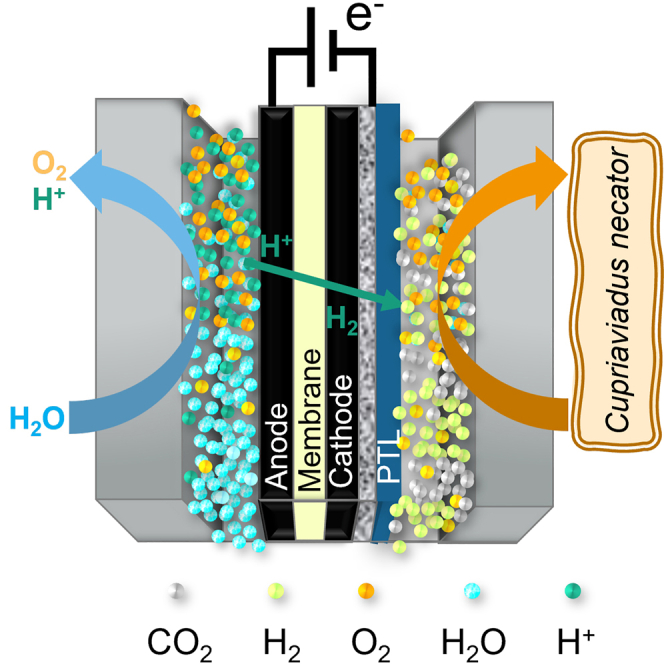

## Introduction

The application of microbial systems in biotechnology presents a promising path for advancing green chemistry. In this regard, the H_2_-oxidizing bacterium *Cupriavidus necator* has attracted considerable attention due to its metabolic versatility, rapid growth rate (up to 0.42 h^−1^),^1^ and its natural ability to accumulate the plastic-like biopolymer polyhydroxybutyrate (PHB) up to 80% of its cell dry weight (CDW).^2^ *C. necator* harnesses the reducing power of H_2_ through its native [NiFe]-hydrogenases, which regenerate NADH directly and ATP indirectly via a proton-motive force with O_2_ as terminal electron acceptor.^3^ In turn, these two cofactors drive the Calvin-Benson-Bassham (CBB) cycle, converting CO_2_ into biomass.^3^^,^^4^^,^^5^ The entire genome of *C. necator* has been sequenced,^6^ giving rise to detailed metabolic characterization,^4^^,^^7^ and molecular genetic engineering strategies.^8^^,^^9^ This groundwork has empowered scientists to harness *C. necator* for atom-efficient CO_2_ bioconversions driven by H_2_ and O_2_,^10^ even surpassing the efficiency of plants.^11^^,^^12^ As such, chemolithoautotrophic cultivation of *C. necator* can provide a biosynthetic CO_2_ assimilating alternative to the synthesis of chemicals that are conventionally derived from fossil sources. One such example is isopropanol (IPA), which serves as an effective drop-in fuel, while also being widely utilized as a solvent, disinfectant, and polymer precursor.^13^ Conventional large-scale production still relies on energy-intensive cracking or reforming of propane.^13^ Metabolic pathways have been developed for the production of potential advanced biofuels by microorganisms to reduce carbon emissions.^14^ *C. necator* was previously engineered for heterologous IPA synthesis by deleting the native PHB biosynthetic pathway yielding strain *Re*2133 (*ΔphaB1B2B3C1*)^15^ and substituting it with an isopropanol production pathway contained on plasmid.^16^ PHB acts as an energy storage and reservoir for excess sequestered carbon under unfavorable growth conditions, when cell growth slows down but central carbon metabolism continues.^17^^,^^18^ Its precursor, acetyl-CoA, can thus be accumulated by typical nutrient limitations like phosphate, O_2_, sulfur, or nitrogen.^2^^,^^19^^,^^20^ The latter is leveraged to regulate acetyl-CoA accumulation in *Re*2133 in lab cultivations.^16^^,^^21^^,^^22^ The *N*-limitation triggered the accumulation of pyruvate, the direct precursor of acetyl-CoA, as has been shown in *Re*2133.^21^ For the downstream conversion of acetyl-CoA to IPA, the native β-ketothiolase (PhaA) and acetoacetyl-CoA-transferase (CtfAB) were overproduced. Codon-optimized acetoacetate decarboxylase and alcohol dehydrogenase from *Clostridium* sp. were heterologously produced to convert acetoacetate to acetone and finally to IPA. The resulting pathway (Figure S1) can be expressed constitutively using a P_Tac_ promoter (*Re*2133/pEG7b), or in a growth-decoupled manner using an arabinose-inducible P_Bad_ promoter (*Re*2133/pEG7c). The published performance of these strains under various conditions have been summarized in Table 1. Both strains have previously demonstrated the biosynthesis of IPA from solely CO_2_, H_2_ and O_2_.^22^

**Table 1:** Key performance indicators of published cultivations with *Re*2133 to produce IPA

Growth mode	Plasmid	*μ*_max_ [h^−1^]	*C*_IPA_^a^ [gL^−1^]	CDW [gL^−1^]	Time [h]	*q*_overall_ [gg^−1^h^−1^]	*q*_max_ [gg^−1^h^−1^]	*Y*_overall_ [gg^−1^]	*Y*_max_ [gg^−1^]	Ref. #
Heterotrophic	pEG7c	0.17	3.44	0.82	96	0.044	0.093	0.16	0.21	Grousseau et al.^16^
Heterotrophic	pEG7b	0.06	2.27	1.99	88	0.03	0.085	0.12	ND	Grousseau et al.^16^; Garrigues et al.^22^
Heterotrophic drip-feed	pEG7c	0.22	8.5	17	70	0.0071	0.036	0.159	0.159	Marc et al.^21^
Autotrophic, pressure	pEG7b	0.05	3	2.9	85	0.013	0.053	0.087	ND	Garrigues et al.^22^
Autotrophic continuous	pBBR-IPA-4	ND	7.7	ND	475	ND	ND	0.278	0.278	Bommareddy et al.^10^
Autotrophic with *in situ* electrolysis, CoPi	pEG12	0.08	0.216	0.8	120	ND	ND	ND	ND	Torella et al.^23^
Autotrophic with *in situ* electrolysis, CoPi	pEG12	0.08	0.6	0.6^b^	144	ND	ND	ND	ND	Liu et al.^12^
Autotrophic with *in situ* electrolysis, pentlandite	pEG7b	0.07	0.65	1	170	0.0038	ND	0.054	ND	This work
Autotrophic with *in situ* electrolysis, pentlandite catalyst, O_2_ limitation	pEG7c	0.08	1.1	0.89	400	0.0028	ND	0.0086	ND	This work

ND = no data.

a In solution only, disregarding any evaporated isopropanol.

b CDW were estimated based on OD_600_ and calibration curve in Figure S2C.

However, mixtures of H_2_ and O_2_ are explosive in a wide range of compositions (4–75% H_2_ in air)^24^ The gas composition required for optimal chemolithoautotrophic growth (7:2:1 H_2_:O_2_:CO_2_)^25^^,^^26^ is therefore highly explosive, which poses a serious safety concern especially at large scales and elevated pressures required to attain high cell densities.^22^ Furthermore, high pressure or intense mixing is required to overcome the poor solubility of H_2_ in water. To address these challenges, adapting gas supply to the microbial demand can minimize losses and reduce the need for pressurization, allowing for more controlled cultivation conditions.^27^ The supply of H_2_ and O_2_ can be easily regulated by tuning the electrolysis of water. Such electrochemical approaches to drive biological or chemical transformations have gained significant interest due to their tunability, sustainability, and compatibility with renewable energy sources.^28^^,^^29^^,^^30^ A PEM electrolyzer approach has previously been employed to supply the required gases for gas fermentation, although the water electrolysis step was conducted separately from the microbial culture.^31^ *In situ* electrolysis of water offers a promising avenue for continuously supplying H_2_ and O_2_ as needed, however, doing so directly in the culture medium poses multiple challenges. The electrodes may harm *C. necator* by perforating the outer membrane at high potentials, leaching of toxic elements, or formation of reactive oxygen species (ROS).^11^^,^^32^ In turn, *C. necator* may harm the electrodes as well, either by directly degrading the electrode material, or by formation of biofilms that lead to fouling and reduced electrode performance.^33^ All the while, the electrolyzer needs to robustly retain electrochemical efficiency irrespective of the growth medium used as electrolyte. For example, biocompatible CoPi cathodes have been developed to produce H_2_ directly in the presence of *C. necator* while mitigating toxicity and maintaining electrode integrity.^12^ Simplifying compatibility considerations, a proton exchange membrane (PEM) enables separate anolyte and catholyte compartments, allowing the culture medium to serve as the catholyte while the anolyte can be regular water or a buffer solution.

The incorporation of a zero-gap cell configuration fitted with a pentlandite-type catalyst (Fe_3_Ni_3_Co_3_S_8_) for H_2_ production further enhances biocompatibility as well as electrochemical efficiency.^34^^,^^35^^,^^36^ This technology aims to minimize resistance and ionic losses by bringing the two electrodes in nearest proximity, separated only by a PEM to avoid short-circuit and to supply all protons required for the H_2_ evolving reaction (HER) stoichiometrically from the O_2_ evolving reaction (OER). A key modification in this zero-gap cell design is the partial separation of the cathodic catalyst from microbial processes using a porous transport layer (PTL), comprising a conductive metal mesh hot-pressed onto a hydrophobic PTFE membrane. This facilitates efficient H_2_ transport from the cathode to the catholyte, which is recirculated to the bioreactor. Furthermore, pentlandites are a naturally occurring innocuous mineral that is entirely composed of iron, nickel and sulfur,^37^ which are the same exact elements contained in hydrogenases of *C. necator*, specifically as [NiFe] active sites for H_2_ splitting and [FeS] clusters for electron transport.^3^^,^^37^^,^^38^^,^^39^ Pentlandite is chemically compatible with aqueous environments and electrochemically efficient.^37^ The resulting electrolyzer has been successfully demonstrated for methane production using wastewater as the catholyte, enriching a natural consortium of methanogens that directly feed on dissolved H_2_ produced at the cathode.^40^ This electrolyzer has been described in detail, attaining a Faradaic efficiency for the production of H_2_ of 85%.^40^ The aim of this work is to apply the aforementioned zero-gap electrolyzer for H_2_ regeneration in a pure culture of bacteria that have been genetically modified for IPA production, to improve process safety compared to pressurized gas fermentations^22^ while retaining efficiency. Dissolved gas concentrations were continuously monitored to study the interplay between the H_2_ and O_2_ supply of the electrolyzer, bacterial growth and IPA production. Off-gas compositions were analyzed to assess safety hazards. Lastly, the long-term stability of the catalyst was assessed by quantification of minerals on the cathode and in the medium after prolonged operation. This study demonstrates the feasibility of coupling H_2_ oxidizing bacteria with a zero-gap electrolyzer, while studying the interplay of microbial consumption and electrochemical production of gases. To our best knowledge, this work is the first demonstration of PEM-based electrolysis using culture broth-derived catholyte for biosynthesis by a genetically modified bacterium. The utility of this system extends to the safe cultivation of any H_2_ oxidizing bacteria.

## Results and discussion

### Development of a BES for isopropanol production

**Figure 1:**
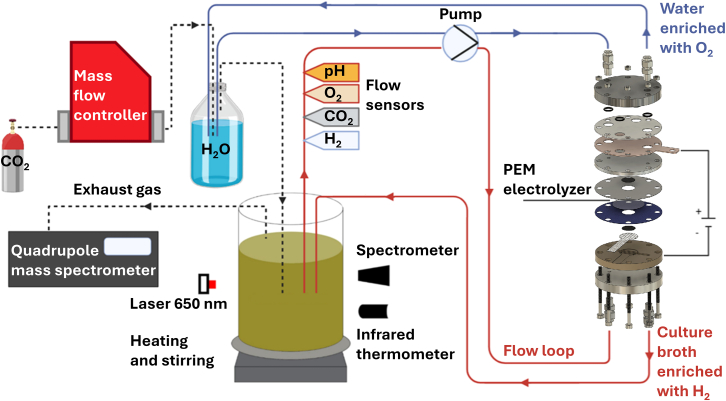
Bioelectrochemical system for *in vivo* isopropanol production The system consists of two flowing loops connected to the zero-gap PEM electrolyzer: the catholyte loop (red) that is continuously enriched with H_2_, and the anolyte loop (blue), continuously being enriched with O_2_. H_2_ evolution reaction (HER) occurs in the catholyte loop, where pentlandite serves as the catalyst. A mass flow controller regulates CO_2_ input, humidified in the anolyte loop and combined with O_2_ from the OER before being supplied to the catholyte for microbial cultivation. A Chi.Bio mini fermenter regulates temperature (30°C), stirring, and optical density (650 nm). A quadrupole mass spectrometer continuously analyzes the exhaust gas. Dotted lines represent gas lines and enclosed lines liquid lines.

A bioelectrochemical system (BES) was developed to investigate the synergy between microbial growth and *in situ* H_2_ production using electrolysis of H_2_O in culture broth for safe and efficient IPA production with recombinant *C. necator* cultures (Figure 1). This BES reactor setup is an extension of the low-cost open source Chi.Bio mini-fermenter platform,^41^^,^^42^ enabling an initial small-scale proof-of-concept. On-site production of H_2_ by water electrolysis significantly reduces the risks associated with handling copious quantities of explosive H_2_. Designing this BES platform offers the advantage of on-demand H_2_ production and the potential for highly parallelized, affordable cultivations on the Chi.Bio platform, thereby increasing throughput for studies of electro-driven cultivations.

H_2_ production occurred through a PEM electrolyzer, featuring spray-coated catalysts, using pentlandite (Fe_3_Ni_3_Co_3_S_8_) for the cathode and IrO_2_ for the anode. Proton diffusion across the PEM and H_2_ diffusion through the back of gas diffusion electrodes (GDEs)—porous materials designed to facilitate gas diffusion—enabled immediate consumption of H_2_ by the cells in the catholyte. This arrangement minimized the risk of gas crossover between the anolyte and catholyte, enhancing the system’s safety. A VMP3 multichannel potentiostat (BioLogic) was used for H_2_ generation via electrolysis. For our experiments, the PEM electrolyzer operated at a current density between 15 and 100 mA cm^−2^ across a 2 cm^2^ electrode area. The flow of CO_2_ gas was adapted to contribute 10% of the total calculated gas supply to the liquid, based on an electrochemical efficiency of 85%.^40^ Gas-impermeable tubing was used throughout the system to minimize gas losses and ensure accurate measurements. To minimize the explosion hazard,^43^ the headspace was not pressurized and instead freely expelled into the fume hood. At the maximal theoretical H_2_ production rate at the highest operating current across cultivations of 60 mA, the BES would only expel 0.355 mL min^−1^ of H_2_. The exhausted H_2_ quickly diffuses in the 2 m^3^ fume hood, which has an air change rate (ACH) of 100 h^−1^, yielding a negligible steady-state H_2_ partial pressure well below 1 ppm. Even in case of a failure of the fume hood, the H_2_ partial pressure stays well below 1 ppm, provided that the laboratory room has a volume of 74800 L and an ACH of 6 h^−1^. As the 1 ppm partial pressure is nowhere near the 4% flammability limit of H_2_, there is no risk of a explosion.^24^ However, preliminary cultivations in our BES (Figures S4 and S5) indicated explosive conditions in the headspace with both O_2_ and H_2_ considerably above the 5% explosion limit.^24^ To ensure safety, the unpressurised headspace has been kept below 1 mL, limiting the maximum energy of a potential oxyhydrogen explosion to just 4 joules, and the fume cupboard pull-down screen provides additional protection. For small-scale reactions this explosion risk is still acceptable, but upscaling may necessitate additional safety measures. Examples of such safety measures are the usage of explosion safe fermenters,^22^ tuning H_2_ production to the bacterial consumption to mitigate excess H_2_ escaping from the liquid phase,^31^ or N_2_-flushing of the headspace to dilute explosive gas mixtures.

In hybrid BESs, selecting suitable materials for both electrochemical and biological performance is critical. Electrolytes that simultaneously support hydrogen evolution reactions (HER) and biological media often diverge from industrial HER conditions, necessitating catalysts that can endure neutral pH and the presence of sulfidic compounds, which are detrimental to platinum, the typical HER catalyst. Non-precious catalysts such as cobalt phosphide (CoP), molybdenum disulfide (MoS_2_), and pentlandite (Fe_3_Ni_3_Co_3_S_8_) offer promising alternatives, with the latter showing particular promise due to its stability and efficiency in poison-laden environments.^40^ The pentlandite catalyst in our BES setup, combined with a porous transport layer (PTL) above the cathode, enables efficient H_2_ production and reduces catalyst exposure to culture broth, enhancing long-term stability. Ion migration through the PTL was observed, affirming the robustness of this design. Comparative experiments, further demonstrated that BES setups without the PTL or using platinum catalysts yielded lower current densities, underscoring the effectiveness of our approach.^40^ This configuration also reduces catalyst costs, supporting the economic viability of large-scale BES applications.

### Isopropanol production from CO_2_ and electricity

Using the developed BES, strains of IPA producing *C. necator* were cultivated on CO_2_ and electricity to study IPA production and resolve process bottlenecks. Precultures of *Re*2133/pEG7b and *Re*2133/pEG7c were prepared in hermetically sealed serum bottles containing MIT media with a gas mixture of 70% H_2_, 20% O_2_ and 10% CO_2_, as this composition was reported to be optimal for chemolithoautotrophic growth.^1^^,^^3^ As follows from Henry’s law, at equilibrium, the partial pressure of a gas in the headspace is directly proportional to its concentration in solution. Therefore, partial pressures of gas mixtures at equilibrium directly translate to %_saturation_ of those gases in liquid, provided consumption is negligible (at low OD). Any deviations from 7:2:1 in %_saturation_ inform how to change the gas flows to attain optimal growth. To reach the same ideal gas composition as the precultures, we tuned the current and CO_2_ flow to achieve dissolved gas saturation levels of 70% H_2_, 20% O_2_, and 10% CO_2_.^27^^,^^31^

**Figure 2:**
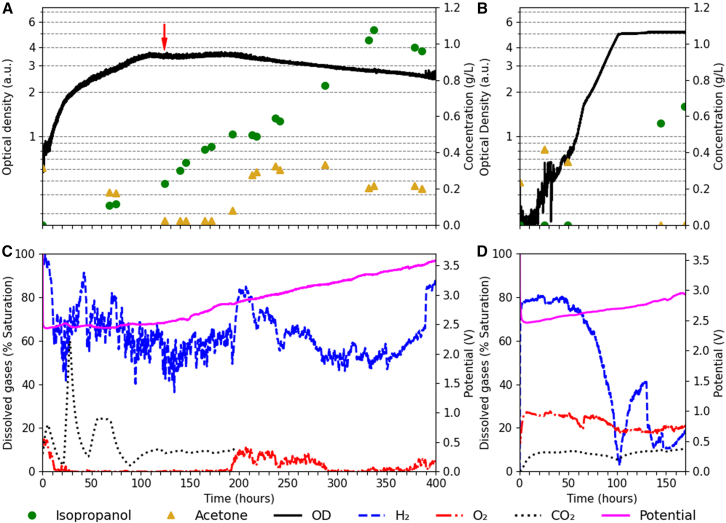
Electro-driven cultivations (A–D) Cultivation of *Re*2133/pEG7c with inducible promoter at 15 mA cm^−2^ with 0.1 mL min^−1^ CO_2_ (A, C) and *Re*2133/pEG7b with constitutive promoter at 30 mA cm^−2^ with 0.2 mL min^−1^ CO_2_ (B, D). The red arrow indicates the moment of induction (A). The optical density was measured at 600 nm and plotted on a semi-logarithmic scale to visualize the exponential growth (A and B).

The inducible strain *Re*2133/pEG7c was cultivated in our BES, with the electrolysis cell providing a constant current density of 15 mA cm^−2^. The MFC was set to a CO_2_ flow rate of 0.1 mL min^−1^ to attain an optimal dissolved CO_2_ concentration (Figures 2A and 2C). The current and CO_2_ flow were set to modest values to mitigate gas losses, improving efficiency. The culture was induced with 0.1 w/v % arabinose at 124 h. By the end of the experiment, the overall integrated gas consumption on the MFC indicated only 2.15 L of CO_2_ was used, corresponding to an effective flow rate of 0.09 mL min^−1^. The maximal titer of isopropanol was reached after 350 h and amounted to 1.1 g L^−1^. Afterward, the IPA concentration declined, and the cultivation was stopped at 400 h. As correction for isopropanol evaporation was not consistently applied across references—and the effect is minor (e.g., 3.5 g L^−1^ versus 3 g L^−1^ in previous work),^22^ reported values do not account for evaporation. Although the dissolved gases were initially close to optimal and no lag-phase occurred, the comparatively low current density caused the dissolved O_2_ to inadvertently become limiting between 20 and 190 h, leading to biphasic exponential growth and a μ_max_ of 0.08 h^−1^ (Table 1). In other cultivations, we addressed this by increasing the current density to 30 mA cm^−2^ (Figure S4), or by on-demand addition of O_2_ with a separate MFC (Figure S5). This alleviated the O_2_ limitation and yielded a μ_max_ of 0.19 h^−1^. However, cultivations that were not O_2_ limited yielded up to 0.05 g L^−1^ IPA (Figures S4 and S5). The observed low product titers under O_2_ unlimited conditions were independently confirmed in batch cultivations of *Re*2133/pEG7c (Figure S6B). The final CDW of 0.89 g L^−1^ exceeded the reported values for electro-driven isopropanol production in *C. necator* (Table 1), highlighting the biocompatibility of the integrated PEM electrolyzer. The pronounced fluctuations in dissolved CO_2_ outside the operating range (0–25%_saturation_) eventually led to the loss of sensor signal observed at 200 h. There was a significant carry-over of the acetone precursor from the precultures, but no IPA detected in the first sample. When the cultivation attained a maximal OD of 3.8 in Chi.Bio, the nitrogen was depleted and the growth abruptly plateaued. The product formation was also recalculated in more industrially relevant key performance indicators (KPIs), overall yield and overall specific productivity. Overall specific yield reports product formation per substrate consumed. It amounted to 0.0086 g_IPA_ g_CO2_^−1^, or 0.0129 carbon mol_IPA_ carbon mol ^−1^_CO2_ for this cultivation (Table 1). The latter unit indicates carbon mole product per carbon mole substrate and corrects for the net stoichiometry of the reaction to purely reflect how many moles of carbon atoms are converted from substrate to product. Overall specific productivity can be interpreted as the overall yield corrected for the time it takes to attain that yield. For this cultivation, the overall specific productivity reached 0.0028 g_IPA_ g_CO2_^−1^ h^−1^ (Table 1). We attribute the comparatively low yield and productivity to our system being open as opposed to closed, which incurs losses for exhausted gases. In addition, delayed precursor formation extended the cultivation period required to reach maximum titer and contributed to the high cumulative CO_2_ consumption, reducing yield and productivity. As the exhaust gas contained up to 85% H_2_ (Figure S11), this negatively impacted the Faradaic efficiency for the production of isopropanol. This value represents the fraction of the applied current that ends up in IPA (Data S1) and reached 2.0% for this cultivation. Faradaic efficiency could be improved by operating the system in a closed configuration. To monitor the stability of the electrolyzer during prolonged operation with culture broth as catholyte, the potential required to maintain a constant current density was plotted (Figure 2A). During the cultivation, the required potential to maintain a constant current density increased from 2.4 V to 2.8 V over the first 170 h, and up to 3.6 V at 400 h, after which the electrolysis cell was not reusable. Due to the unpressurized exhaust, low CDW and small-scale of the cultivation (making gaseous bubbles relatively large, and the time spent in the culture broth relatively short), preliminary experiments pointed out that the off-gas composition did not fluctuate throughout the cultivation (Figures S4 and S11). To minimize unsupervised exposure of the MS capillary to an exhaust that could accidentally expel liquid from a full reactor, we determined the gas composition on supervised sampling times henceforth. As we cannot hermetically seal the MS capillary to the exhaust, there is an inadvertent mixing with air, reflected in ∼30% N_2_ component. Off-gas composition was therefore also reported corrected for air leakage (Figure S11). with the addition of 0.09 mL min^−1^ CO_2_.

Subsequently, the constitutive strain *Re*2133/pEG7b was cultivated in the BES. The electrolysis cell provided a constant current density of 30 mA cm^−2^. The CO_2_ MFC was set to a corresponding flow rate of 0.2 mL min^−1^, effectively delivering the same gas mixture as previously with twice the flow rate to avoid gas limitation (Figures 2B and 2D). Upon equilibration, initial levels of dissolved gases corresponded to a near-optimal supply of H_2_: O_2_: CO_2_ (Figure 2D). During growth, the gas consumption increased with cell density, mostly affecting the dissolved H_2_ concentration. This is to be expected, as H_2_ is consumed in a 2.71 : 1 stoichiometry with O_2_ for respiration and a 7.77 : 1 stoichiometry with CO_2_ for biomass formation.^44^ Furthermore, H_2_ is least soluble making fluctuations expressed in %_saturation_ more pronounced. The maximum specific growth rate reached 0.07 h^−1^, slightly higher than previously reported for autotrophic and heterotrophic cultivations (Table 1). The final CDW of 1 g L^−1^ is the highest reported for electro-driven isopropanol production in *C. necator* (Table 1). As previously observed (Figure 2A), a substantial amount of acetone precursor was carried over from the precultures. Although this precursor continued to accumulate during growth, no initial IPA was detected. At an OD of 5.2 (corresponding to CDW of 1 g L^−1^) the cultivation reached the nitrogen depleted IPA production, with detectable levels of dissolved H_2_ (minimally 1.6%_saturation_). Nitrogen limitation resulted in plateauing growth, accompanied by reduced H_2_ consumption. However, even without further growth, the cultivation still consumed some H_2_ for IPA production and maintenance metabolism. The cultivation was stopped at 170 h when the culture converted all acetone to IPA, to a final titer of 0.65 g L^−1^. When normalized to CDW, this titer is half of what was achieved in autotrophic batch cultivation of this strain, accumulating up to 1.3 g L^−1^ IPA per gram biomass (Figure S6A). For this cultivation, the specific productivity reached 0.0038 g_IPA_ g_CO2_^−1^ h^−1^ and the specific yield reached 0.054 g_IPA_ g_CO2_^−1^ or 0.081 C mole_IPA_ C mole_CO2_^−1^. The overall Faradaic efficiency for the production of IPA was 1.96%. Comparing our open system to published pressurized autotrophic conditions, the yield and productivity for this strain were 1.6-fold and 3.4-fold lower in our experiments (Table 1). However, due to challenges in reproducing the gas limitation that was instrumental in the performance of *Re*2133/pEG7c, we hesitate to declare one or the other strain as categorically superior to this BES. Over the 170-h cultivation, the required potential to maintain a constant current density at 15 mA cm^−2^ increased slightly from 2.4 V to 2.9 V, indicating mild deterioration of the electrolysis cell. In a separate abiotic experiment, the dissolved H_2_ concentrations were monitored during electrolysis from 0 to equilibrium. The resulting k_L_a for H_2_ mass transfer in our reactor setup was determined to be 1.33 h^−1^ (Figure S7), which is reasonably low, considering the unpressurized open system and small reactor volume.^45^

### Stability of the pentlandite-catalyst cathode

The abiotic electrochemical tests highlighted the stability and material compatibility of the pentlandite-based cathode under various fixed currents (30 and 40 mA cm^−2^ respectively), with a voltage maintained between 2.0 and 2.1 V throughout one-day operation (Figure S8). All biotic experiments exhibited a steady, albeit slow, increase of potential over time in absence of biofilm formation on the cathode surface (Figure 4) or inside tubes. The observed deterioration was more extreme compared to published work with wastewater as catholyte, where upwards of 250 days of continued operation were achieved.^40^ This behavior can likely be explained by two key factors: loss of the cathode material that catalyzes electrolysis (Fe_3_Ni_3_Co_3_S_8_) and the accumulation of precipitates from the catholyte, such as struvite,^46^ which obstruct catalytic sites.

**Figure 3:**
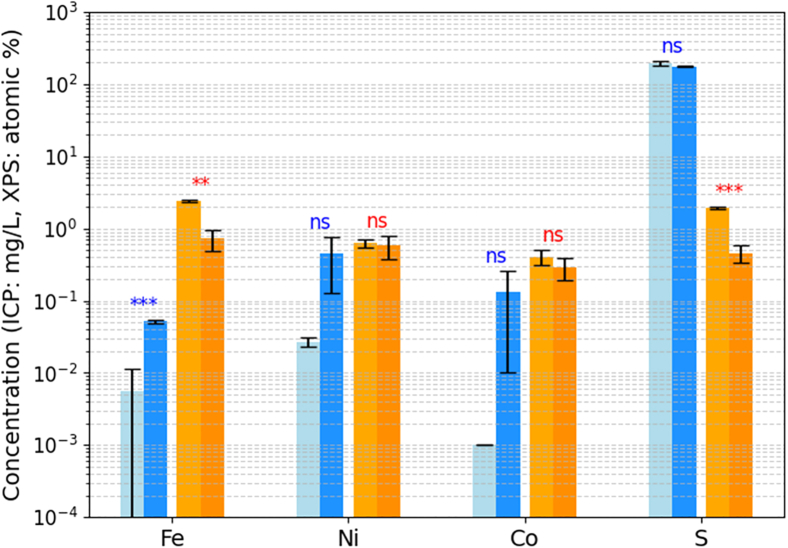
ICP-OES analysis of media and surface XPS of pentlandite cathode, before and after usage ICP-OES analysis was performed in fresh media and media following chronopotentiometry at fixed current density of 60 mA cm^−2^ in abiotic experiments. Blue bars pertain to ICP-OES of the media (light blue: fresh media; dark blue: media after electrolysis). Orange bars pertain to surface XPS of the electrodes (light orange: fresh electrodes; dark orange: postmortem electrodes). Error bars represent standard deviations of triplicates, ICP-OES on fresh media was performed in duplicate. The significance of the difference between fresh and used media for each element is indicated above the bars (∗∗: *p*-value >0.01, ∗∗∗: *p*-value >0.001, ns: not significant).

Post-electrolysis ICP-OES analysis (Figure 3) indicated an accumulation of Co in the medium after electrolysis, which was detected only in traces in the MIT medium as Co is not a medium component (Table S1). This observation suggests the occurrence of catalyst leaching into the medium during electrolysis. Furthermore, other catalyst components Fe and Ni were also detected at elevated concentrations in the used medium. Toxicity tests showed that the experimentally determined concentrations of leached elements had no inhibitory effect on the growth of *C. necator* (Figure S12).

**Figure 4:**
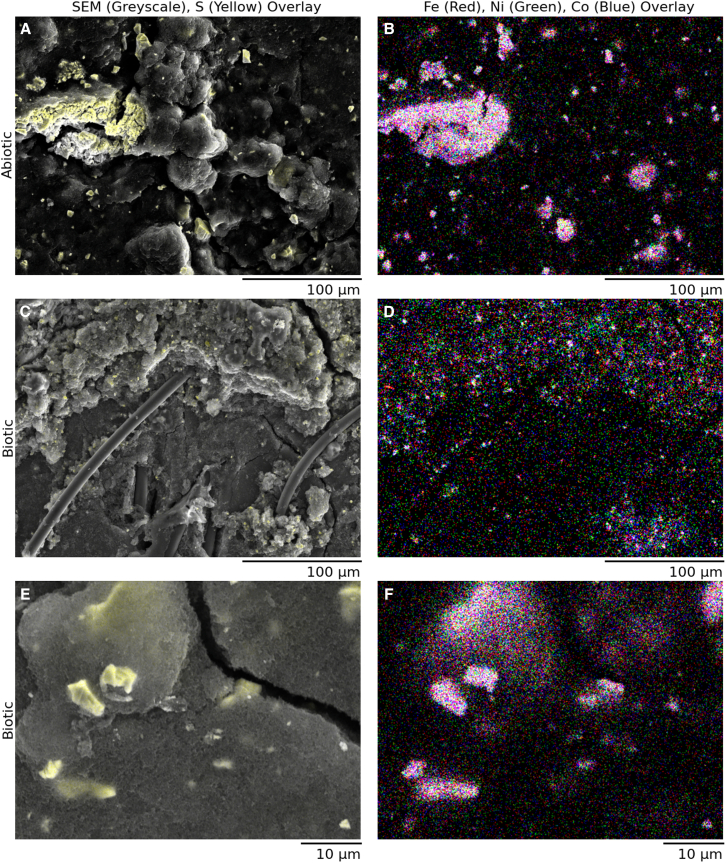
Scanning Electron Microscopy (SEM) and Energy Dispersive (A–F) X-ray (EDX) analysis of pentlandite (Fe_3_Ni_3_Co_3_S_8_) electrodes under abiotic and biotic conditions Greyscale SEM images (A, C, E) are overlaid with EDX sulfur (yellow) to depict sulfur distribution on the electrode surface, while EDX maps (B, D, F) show the elemental distributions of iron (red), nickel (green), and cobalt (blue). The top row shows abiotic electrodes at a 100 μm scale, the middle row presents biotic electrodes at a 100 μm scale, and the bottom row shows biotic electrodes again, at a 10 μm scale to verify the absence of a biofilm. A technical replicate of these images is shown in the supplement (Figure S10).

Surface XPS analysis of the electrodes reveals multiple mechanisms contributing to the observed potential drop although no biofilm formation of *C. necator* on the cathode was detected (Figure 4). Notable elemental accumulations of O, N, P, Ca, Na, and Mg were identified which are coming from the buffer solution in the catholyte (Figure S9), alongside a decrease in pentlandite catalyst content (Figure 3). Energy Dispersive X-ray Spectroscopy (EDX) analysis confirms the presence of pentlandite crystals, with a uniform distribution of metal oxides across the electrode surface (Figures 4 and S10). This accumulation is consistent with the transport of S^2−^ and PO_4_^3−^ ions from the catholyte to the anolyte via the PTL and cathode before reaching the PEM.

Despite material loss over extended cycles, the results demonstrate the high stability of pentlandite, supporting its suitability for long-term use in BES. To support higher cell density cultures of *C. necator* (>100 g L^−1^),^47^ the pressure or the operation current could be increased, striking a compromise between electrode stability and microbial H_2_ demand. Conversely, H_2_ losses could be minimized by developing a closed system, necessitating dynamic tuning of electrolysis to avoid internal leakage due to high pressure on the PEM. Complementary ICP-OES analysis suggests that while pentlandite offers initial robustness, further optimization to reduce leaching could enhance BES performance. Interestingly, the leached species also contribute essential minerals to the MIT medium, potentially replenishing metal ions during prolonged cultivation. This interplay underscores a balance between catalyst stability and metal ion availability in BES applications. The pentlandite-type catalyst (Fe_3_Ni_3_Co_3_S_8_) demonstrates superior robustness compared to noble metals like platinum, which has shown limited stability in similar systems due to poisoning by the formation of compounds like H_2_S and CO. Additionally, the use of pentlandite avoids the toxicity and high cost associated with platinum, making it a more scalable and practical choice for culture broth-based BESs.^38^

### Conclusion and limitations of the study

This study demonstrates the feasibility of integrating a PEM electrolyzer with microbial cultivations for bioelectrosynthesis of isopropanol from CO_2_ and electricity. The setup achieved a high degree of biocompatibility and enabled *in situ* H_2_ supply under safe, small-scale conditions, validating its potential as a platform for electro-driven microbial production. The modular BES design allowed flexible control of gas inputs and electrolysis conditions and supported growth and isopropanol formation in recombinant *C. necator* strains. In contrast to other BES designs that employ more conventional noble metal catalysts (e.g., platinum, palladium),^48^ pentlandite provided a cost-effective and biocompatible alternative while maintaining electrochemical performance over prolonged cultivation periods.^37^ Cultivation conditions in the BES could easily be adapted to accommodate other H_2_-oxidizing bacteria (e.g., *Hydrogenophaga pseudoflava*, *Thermoanaerobacter kivui*, and *Paracoccus denitrificans*).^49^^,^^50^^,^^51^

Nevertheless, several aspects of the system remain to be optimized. Current densities and gas flows were deliberately kept low to minimize gas loss and explosion hazards, but this led to O_2_ limitations during extended cultivations. While this was alleviated in later runs by increasing current density or supplementing O_2_ on demand, this reactive control introduces complexity. In addition, the open system design caused significant gas escape, especially H_2_, which negatively impacted Faradaic efficiency and overall carbon utilization. Closed-system operation is likely essential to improve both yield and efficiency.

While the PEM electrolyzer remained operational over extended periods, performance degradation was observed, with increasing voltage requirements and eventual electrode failure after ∼400 h. This suggests long-term fouling or poisoning by culture medium components necessitating future improvements in catalyst longevity, particularly when running at higher operating currents required for high density cultures. Attaining a high cell density is a stepping stone toward improving isopropanol titers, which remained low compared to pressurized reactors presented in literature.^22^

Finally, while safety measures were sufficient for small-scale proof-of-concept, upscaling this system will require redesigned reactor geometries, active gas-phase control, and improved headspace management to safely handle explosive gas mixtures and reduce evaporative or diffusive losses. Larger cultivation vessels need proportionally more electrode surface area for the supply of oxyhydrogen, which could be realized by e.g., electrolyzer stacking. Future work should explore scaling up the BES to e.g., 10 L knallgas fermenter, to evaluate safety and efficiency at technical relevant volumes.

## Resource availability

### Lead contact

Further information and requests for resources should be directed to and will be fulfilled by the lead contact, Lars Lauterbach (lars.lauterbach@iamb.rwth-aachen.de).

### Materials availability

Raw- and metadata generated in this study have been deposited and are publicly available as of the date of publication at Zenodo: https://doi.org/10.5281/zenodo.14224096. This study did not generate new unique reagents.

### Data and code availability

All original code and microscopy data have been deposited at Zenodo: https://doi.org/10.5281/zenodo.14224096 and is publicly available as of the date of publication. Any additional information required to reanalyze the data reported in this paper is available from the lead contact upon request.

## Acknowledgments

This project was funded by the 10.13039/501100001659Deutsche Forschungsgemeinschaft (DFG, German Research Foundation – project number: 445397982) within SPP2240 eBiotech and through the cluster of excellence EXC 2186–390919832 “The Fuel Science Center”. Funding by the Werner Siemens Foundation within the WSS project of the century “catalaix” is acknowledged. Our gratitude extends to Dr. Paul Cordero for the fruitful discussions. We thank Prof. Lars Blank for the use of the HPLC.

## Author contributions

Conceptualization, P.S., S.G., U.P.A., and L.L.; data curation, P.S., R.R., and A.I.; formal analysis, P.S. and A.I.; investigation, P.S., R.R., A.I., and I.W.; methodology, P.S., R.R., and A.I.; software, P.S.; project administration, P.S. and R.R.; validation, P.S. and R.R.; visualization, P.S.; writing – original draft, P.S., R.R., and I.W.; writing – review and editing, P.S., R.R., A.I., I.W., S.G., U.P.A., and L.L.; funding acquisition, U.P.A. and L.L.; resources, I.W., S.G., U.P.A., and L.L.; supervision, U.P.A. and L.L.

## Declaration of interests

The authors declare no competing interests.

## STAR★Methods

### Key resources table

**Table:** 

REAGENT or RESOURCE	SOURCE	IDENTIFIER
**Bacterial and virus strains**		

*C. necator* H16 *Re*2133/pEG7b: Δ*phaB1B2B3* Δ*phaC,* containing plasmid with *thl*, *ctf*, *adc* and *adh* under the control of P_TAC_	INSA Toulouse	ATCC: 17699 (ancestor)
*C. necator* H16 *Re*2133/pEG7c: Δ*phaB1B2B3* Δ*phaC,* containing plasmid with *thl*, *ctf*, *adc* and *adh* under the control of P_BAD_	INSA Toulouse	ATCC: 17699 (ancestor)

**Chemicals, peptides, and recombinant proteins**		

CaCl_2_ · 2 H_2_O	Roth	T885.2
K_2_SO_4_	Roth	X889.1
MgSO_4_ · 7H_2_O	Roth	P027.2
Na_2_HPO_4_ · 12H_2_O	Roth	T106.2
NaH_2_PO_4_ · 2H_2_O	Roth	T879.2
NH_4_Cl	Roth	K298.2
NiCl_2_ · 6H_2_O	Roth	7312.3
FeSO_4_ · 7H_2_O	Roth	3722.1
MnSO_4_ · H_2_O	Roth	X890.1
ZnSO_4_ · 7H_2_O	Roth	T884.1
CuSO_4_ · 5H_2_O	Roth	P025.1

**Deposited data**		

Raw data	This work	Zenodo: https://doi.org/10.5281/zenodo.14224096

**Software and algorithms**		

EC-lab®	BioLogic	V11.43
PV Mass Spec 64- bit	Pfeiffer Vacuum	V23.06.00-c
Getred-y	Vögtlin	Version 5 5.7.1.3
PreSens Measurement Studio	PreSens	V2
SensorTrace Logger	Unisense	v3.4.400.20899
Chromeleon	ThermoFisher	v7.2.10
CasaXPS	Casa Software	Version 2.3.15

### Experimental model and subject details

*Cupriavidus necator* strain *Re*2133 was used for all experiments. This strain is a derivative of H16 engineered to lack polyhydroxybutyrate (PHB) biosynthesis and enable heterologous isopropanol (IPA) production. Cells were cultivated under autotrophic conditions with CO_2_ as the sole carbon source, using H_2_ and O_2_ as electron donor and acceptor, respectively. Two engineered plasmids, pEG7b and pEG7c, were used for IPA production in *C. necator Re*2133.^16^^,^^21^^,^^22^ Plasmid pEG7b contains a constitutive P_Tac_ promoter, while pEG7c has an arabinose-inducible P_Bad_ promoter. Both plasmids harbor codon-optimized *adh* and *adc* genes from *Clostridium*, enabling the conversion of pyruvate (derived from the CO_2_-fixing CBB-cycle) to IPA. The conversion pathway consists of beta-ketothiolase (THL), acetoacetyl-CoA transferase, (CTF), acetoacetate decarboxyalse (ADC) and alcohol dehydrogenase (ADH) to catalyze the final step from acetone to IPA (Figure S1).

### Method details

#### Chemolithoautotrophic preculture

To stratify the lag phase across inoculations, main cultivations were inoculated from preculture. Thawed glycerol stocks of *Re*2133/pEG7b and *Re*2133/pEG7c were used to inoculate 10 mL MIT medium^16^ containing 0.1 g L^-1^ kanamycin sulfate (Table S1) in a 250 mL gas-tight bottle. The gas environment was established by purging the culture vessel with a gas mixture (10% CO_2_, 20% O_2_, and 70% H_2_) at a rate of 100 mL min^-1^ for five minutes to displace ambient air. Subsequently, the exhaust needle was removed for a further one-minute purge at the same flow rate to attain an overpressure of 1.3 bar. Fresh glycerol stocks were prepared from chemolithoautotrophic precultures prior to each experiment. Aliquots were used only once and not re-frozen after thawing, which was instrumental in preventing a detectable lag-phase. Precultures were grown for two days at 30°C before inoculating the main reactor.

#### Correlation between NH_4_Cl, OD and CDW

To determine the impact of NH_4_Cl concentrations on final cell density, shake flasks containing *Re*2133/pEG7c were cultured in MIT medium supplemented with fructose and varying concentrations of dissolved NH_4_Cl (0.28–2.7 g L^−1^) (Figure S2) at 30°C and 200 rpm for four days. The relationship between NH_4_Cl concentration and optical density (OD) determined in Chi.Bio and in an Ultrospec 10 cell density meter (Biochrom) was derived to inform NH_4_Cl requirement. The NH_4_Cl concentration that corresponded to a final CDW of 1 g L^-1^ was added to the MIT media for cultivation in the bioelectrochemical system (Table S1). To establish a correlation between OD and CDW, *Re*2133/pEG7c was cultivated in MIT medium with fructose across a range of NH_4_Cl concentrations. After five days of growth, triplicate samples (1 mL each) from each flask were centrifuged in Eppendorf tubes, washed with MilliQ water, and the remaining pellet was dried at 60°C for one week. The CDW was then correlated with final OD readings, generating a CDW-to-OD curve (Figure S2).

#### Isopropanol cultivation and sampling

Isopropanol production was achieved using *Re*2133/pEG7b and *Re*2133/pEG7c grown in MIT medium (Table S1) to nitrogen depletion, then induced with 0.1% arabinose if applicable (Figures 2, S4, and S5). Over the ensuing 100-hour production phase, samples were collected daily or bi-daily, centrifuged (20817 x g, 5 min), and the supernatants stored at −20°C for later HPLC analysis. Autotrophic batch cultivations of isopropanol producing *C. necator* strains were conducted in duplicate (duplicates indicated as dotted line) in 1 L baffled Schott bottles closed with 2-valved, hermetic lids (Figure S6). The headspace was filled with a gas mixture of H_2_/O_2_/CO_2_= 70/12/18 mol% with 1.5 bar overpressure. Pressure was measured during cultivations, and the gas phase was replaced when necessary. Cultivations were conducted in 100 mL mineral salt medium containing 1.0 g L^-1^ NH_4_Cl which suffices for a biomass formation of 2 g L^-1^.

#### Bioreactor specifications

The Chi.Bio system^41^^,^^42^ integrates an incubator, shaker, plate reader, and robotic liquid handling into a single automated platform. The control unit is a Beaglebone Black Rev C computer, running on Linux with a Python-based backend and a web-server interface in HTML/Javascript, allowing control of up to eight reactors in parallel via USB or Ethernet. Each reactor accommodates a 30 ml flat-bottom tube and uses a 12V power supply, with connections via micro-USB for communication with the control computer and pumps. Liquid handling is facilitated by four independently controlled peristaltic pumps, with a maximum flow rate of 1.0 mL s^-1^, suitable for OD regulation, chemical induction, media addition, or inter-reactor mixing. Optical outputs include a seven-colour LED array for fluorescence, optogenetics, and photosynthesis, as well as a UV LED and a 650 nm laser for precise optical density measurements, supported by individual current-limiting drivers. Spectrometry capabilities cover a broad wavelength range (410–670 nm) with adjustable gain and integration time, and mechanical aperture control. Temperature monitoring includes air measurement with chip thermometers and media measurement via an infrared thermometer, accurate to within ±0.5°C and ±0.2°C respectively. A resistive plate provides heating, offering a heating rate up to 2.0°C min^-1^. A speed-adjustable fan with magnets enables magnetic stirring, compatible with stir bars up to 15 mm.

#### Electricity-driven cultivation

The Chi.Bio reactor was adapted to accommodate electricity-driven cultivation. A hole of 1 cm in diameter was drilled through the top lid of the Chi.Bio culture vessel. The 30 mL culture vessel flask was fitted with a stirring bar and sealed by fitting a septum on the inside of the drilled cap. This allowed for leak-proof incorporation of a flow-loop to continuously exchange media through cannulas that pierce the septum. The flow loop represents a peristaltically circulated volume exchanging the culture broth between the culture vessel and the cathode flow field of the zero-gap cell, with a total volume of 7.5 mL, including the flow field. The gas composition of the liquid was continuously monitored by in-flow sensors incorporated in the flow loop immediately after the culture vessel, i.e. PreSens in-line optical flow sensors to monitor O_2_, CO_2_, and pH and a Unisense electrochemical based in-line flow sensor to monitor H_2_ concentrations. The peristaltic pump was set to flow at 10 mL min^-1^, corresponding to a residence time of 3.65 minutes in the reactor with a total volume of 36.5 mL. Preliminary experiments demonstrated that at flow rates of 2 mL min^-1^ or higher, the dissolved gas concentrations measured by the in-line sensors accurately represented those in the culture vessel, as the transit time was too short for metabolic activity to cause measurable distortion of the dissolved gas concentrations (Figure S3). The flow loop was sterilized by pumping 70% ethanol for 10 minutes. The ethanol was removed by washing with sterile MIT medium containing antibiotic for another 2 minutes. In an aseptic environment, the Chi.Bio culture vessel was filled with 29 mL sterile MIT medium, minimizing the headspace. The culture vessel was placed in a Chi.Bio mini-fermenter,^42^ which was set to a temperature of 30°C to support chemolithoautotrophic growth of *C. necator*.^3^ A VMP3 multichannel potentiostat (BioLogic) was connected to the PEM electrolyzer for H_2_ generation in the medium. A Bronkhorst mass flow controller (MFC) was used to regulate CO_2_ input (0.012–0.6 mL min^-1^), which was humidified through double distilled water in the anolyte flask. The humidified CO_2_, along with O_2_ recovered from the OER, was introduced into the culture vessel via a needle. This needle was submerged to avoid H_2_ flowing from the culture headspace into the O_2_ rich headspace of the anolyte bottle. The headspace of the culture vessel was vented into a fume hood, and the exhaust gas composition was continuously monitored using a Pfeiffer vacuum mass spectrometer. Cultures of *Re*2133/pEG7b and *Re*2133/pEG7c grew until nitrogen depletion when they entered a 100-hour production phase. HPLC samples were taken throughout the cultivation to assess intermediate- and product formation. The tubing used for the flow-loops and the gas connection between anolyte bottle and culture vessel was hard Neoprene tubing of dimensions 4 x 1 mm (Roth), alternated with soft PharMed® Pump tubing of dimensions 4.49 x 0.85 mm (Saint-Gobain) to enable peristaltic pumping. Polyethylene hose of dimensions 4 x 2 mm (Landefeld) was used for all gas connections.

#### Analytical methods

Off-gas was analyzed using Mass spectrometry (MS) using a GSD 350 O2C mass spectrometer (Pfeiffer Vacuum), with sampling through neoprene tubing (Roth). The MS scanned target masses (H_2_, O_2_, N_2_, CO_2_, acetone, IPA) in the headspace using a 2 cm^3^ sample every 100 seconds (Figure S11). In-flow dissolved gasses were monitored using optical flow-through sensors from PreSens (FTC series) measured O_2_, CO_2_, and pH in real-time. The O_2_ sensor (FTC-PSt3) and CO_2_ sensor (FTC-SU-CD1-09NaCl-S) monitored concentrations within accuracy limits of ±0.4% for O_2_ and ±10% for CO_2_. A Unisense H_2_ microsensor (H_2_-SL-1/8 model) connected to a UniAmp device quantified dissolved H_2_ with a detection limit of 0.3 μM. To quantify the presence of metabolites, including IPA and acetone, High-Performance Liquid Chromatography (HPLC) was performed using an UltiMate 3000 HPLC system (ThermoFisher) equipped with a Metab-AAC column. Samples were filtered (0.22 μm CA membrane) before injection, and analyses were conducted at 0.5 mL min^-1^ and 50°C with HS_2_O_4_ (5 mM) as the mobile phase. The total runtime per sample was 45 minutes. X-ray photoelectron spectroscopy (XPS) analysis was carried out on a Nexsa G2 surface analysis system with a monochromared, micro-focused, focused, high-efficiency Al Kα X-ray source. The analyzer used was a 180°, double-focusing, hemispherical analyzer with a 128-channel detector. A 400 μm spot was analyzed with a pass energy of 200.0 eV and a step size of 0.100 eV. The results were calibrated based on the carbon signal at 284.8 eV and evaluated using CasaXPS software (version 2.3.15).

Inductively Coupled Plasma Optical Emission Spectroscopy (ICP-OES was employed to quantify the elemental composition of the catholyte, reflective of electrode deterioration. Scanning Electron Microscopy (SEM) coupled with energy-dispersive X-ray spectroscopy (EDX) was conducted using a ThermoFisher Helios G4 to simultaneously examine the surface morphology and elemental composition of the catalyst on the cathode electrode.

#### Determination of mass transfer constant

To determine the k_L_a for the mass transfer of H_2_ to the liquid phase, the reactor was filled with MIT-medium, and the dissolved H_2_ concentration was monitored over time. At atmospheric conditions, the H_2_ concentration was near zero. Then, the culture vessel was purged at 10 mL min^-1^ with a controlled gas composition of 7 : 2 : 1, respectively H_2_ : O_2_ : CO_2_. The dissolved H_2_ concentration rose until it equilibrated at 420 μM. The natural logarithm of the difference between the dissolved H_2_ concentration and the concentration at equilibrium was plotted. The slope of the linear regression revealed a k_L_a of 1.33 h^-1^ (Figure S7).

#### Electrochemical characterization

Abiotic electrochemical experiments were conducted to verify the performance of the PEM electrolyzer with MIT medium in absence of microbial consumption. All abiotic experiments were conducted using chronoamperometry with a Potentiostat (VMP3 Multichannel; BioLogic, France) through a fixed current (Figure S8).

#### Determination of metal leaching toxicity

To investigate the potentially toxic effects of metal leaching, *Re*2133/pEG7c was precultured overnight in MIT-medium supplemented with 200 μg mL^-1^ kanamycin and 4 g L^-1^ fructose. The culture was reinoculated in 40 mL fresh medium of the same composition, which was split in 10 mL aliquots over 4 falcon tubes, one corresponding to the control, one for each metal suspected of leaching into the medium as determined by ICP-OES (Figure 3). As the determined dissolved Ni, Fe, and Co concentrations never exceeded 20 μM, the independent toxic effects of these metals were investigated by addition of 20 μM of the respective chloride salts of these metals to an aliquot of *Re*2133/pEG7c (NiCl_2_, FeCl_3_ and CoCl_2_ respectively). The aliquots were split over the first three rows of a 96-wells plate, the control over the first six wells of the first three rows, the aliquots with chloride salt additions over the last six wells of the row of their respective metal.

### Quantification and statistical analysis

Statistical analyses were performed using standard methods implemented in Python and Excel. All statistical parameters, including the number of replicates (n), measures of center and dispersion, and significance values, are provided in the figure legends. Unless otherwise noted, data are presented as mean ± standard deviation. For Figure 3, differences between fresh and post-electrolysis conditions were evaluated using unpaired two-tailed t-tests; significance thresholds are indicated as ∗∗ (*p* < 0.01), ∗∗∗ (*p* < 0.001), or “ns” for not significant. For Figure S12, shaded regions around the average growth curves represent the standard deviation of six biological replicates. Regression analyses for Figures S2 and S7 were performed using linear least-squares fitting; the negative slope of the linear fit in Figure S7 was used to determine the mass transfer coefficient k_L_a. No formal methods were used to assess the assumptions of statistical tests or estimate sample sizes.
