# Supplementary material for: Isopropanol production from carbon dioxide by Cupriavidus necator using a zero-gap cell with culture broth as catholyte
Source: iScience. 2025 Jun 27;28(8):113018. doi: 10.1016/j.isci.2025.113018 (PMC12341536; doi:10.1016/j.isci.2025.113018)
Supplement: Document S1. Figures S1–S12, Table S1, and Data S1 [file mmc1.pdf]

iScience, Volume *28*

Supplemental information

Isopropanol production from carbon dioxide by *Cupriavidus necator* using a zero-gap cell with culture broth as catholyte

Pierre Schoenmakers, Ramineh Rad, Axel Ihl, Isabell Weickardt, Stéphane Guillouet, Ulf Peter Apfel, and Lars Lauterbach

SUPPLEMENTAL ITEMS

Table S1. (related to STAR Methods: Growth Medium Composition) Composition of the MIT growth medium.

To this medium 0.1 g L^{-1} kanamycin sulfate was added

Chemical	Final concentration [mM]
$CaCl_2 \cdot 2H_2O$	0.544
K_2SO_4	2.58
$MgSO_4 \cdot 7H_2O$	3.25
$Na_2HPO_4 \cdot 12H_2O$	32.39
$NaH_2PO_4 \cdot 2H_2O$	33.33
NH_4Cl	18.7
$NiCl_2 \cdot 6H_2O$	0.0008
$FeSO_4 \cdot 7H_2O$	0.0054
$MnSO_4 \cdot H_2O$	0.0142
$ZnSO_4 \cdot 7H_2O$	0.00835
$CuSO_4 \cdot 5H_2O$	0.002

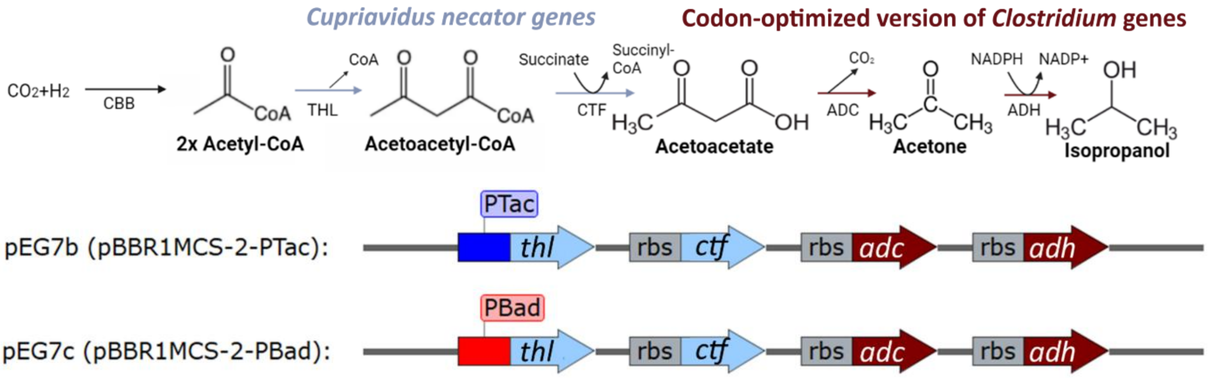

Figure S1. (related to Figure 2) The biochemical pathway of carbon sequestration to isopropanol production (top) and abstract representation of the isopropanol production pathway on plasmids pEG7b and pEG7c (bottom).[1]

The pathway contained on a polycistronic transcript, interspaced by ribosome binding sites (rbs), regulated by either a constitutive P_{Tac} promoter, or an arabinose inducible P_{Bad} promoter. The introduced pathway diverts carbon sequestered in the Calvin cycle to isopropanol. The pathway consists of the native β-ketothiolase (THL), acetoacetyl-CoA-transferase (CTF), and codon-optimized variants of *Clostridium sp.* acetoacetate decarboxylase (ADC) and alcohol dehydrogenase (ADH).

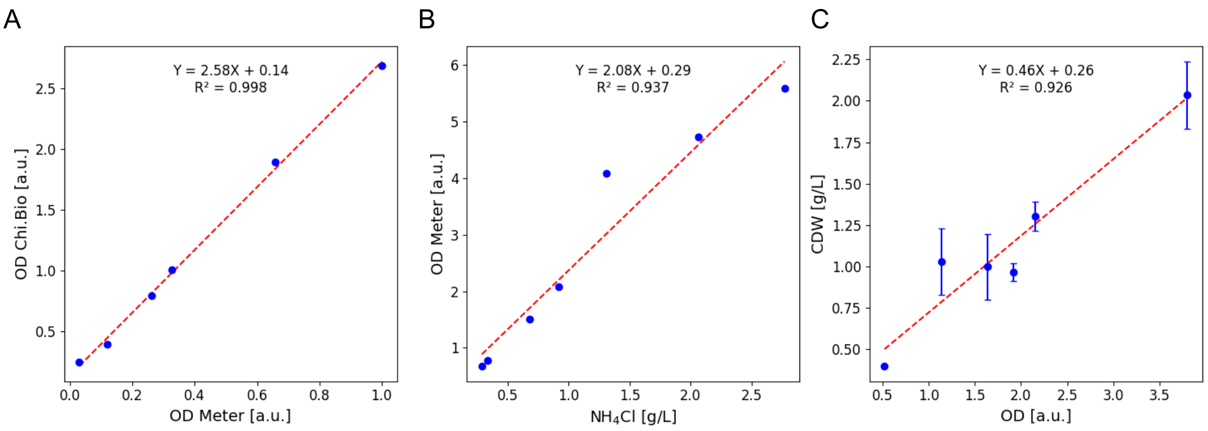

Figure S2. (related to STAR Methods: Correlation between NH4Cl, OD and CDW) Cell density correlations.

The correlation between cell density measured in the OD spectrometer and Chi.Bio (A), the concentration NH_4Cl and the resulting cell density measured in the OD spectrometer (B), and the correlation between cell density measured in the OD meter and the resulting CDW (C).

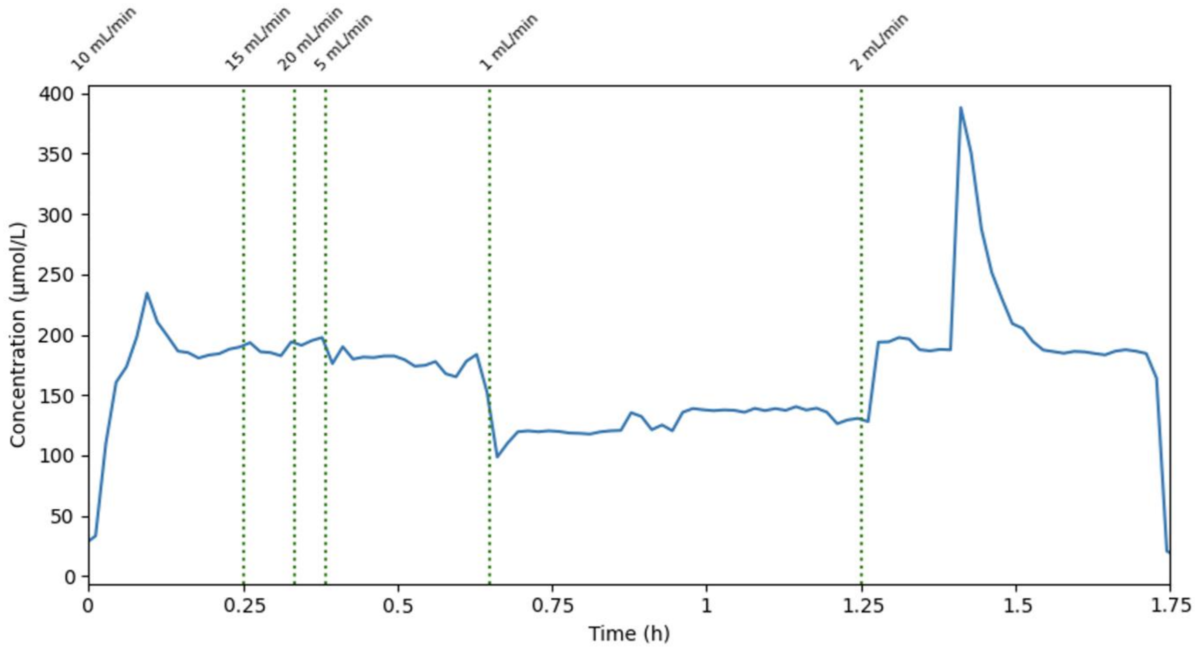

Figure S3. (related to STAR Methods: Electricity-driven cultivation) Effect of pump rate on the measured dissolved H_2 concentration in a flow loop setup.

A *Re*2133/pEG7b culture in the growth phase (CDW = 1 g L^{-1}) was cultivated in the BES setup (Figure 1) under bubbling conditions (H_2, O_2, and CO_2 in a 7:2:1 ratio) at 10 mL min^{-1} without electrolysis. The pump rate was periodically varied, as indicated above the graph. Flow rates of 2 mL min^{-1} or higher show a consistent H_2 concentration across flowrates, ensuring that the measured dissolved gas concentrations accurately reflected those in the culture vessel, whereas at 1 mL min^{-1}, H_2 consumption by the culture in transit from the culture vessel to the sensor led to a measurable reduction in dissolved H_2 concentration. The observed peak at 1.4 h is likely due to a H_2 bubble that temporarily got stuck before the sensor, leading to a locally elevated concentration.

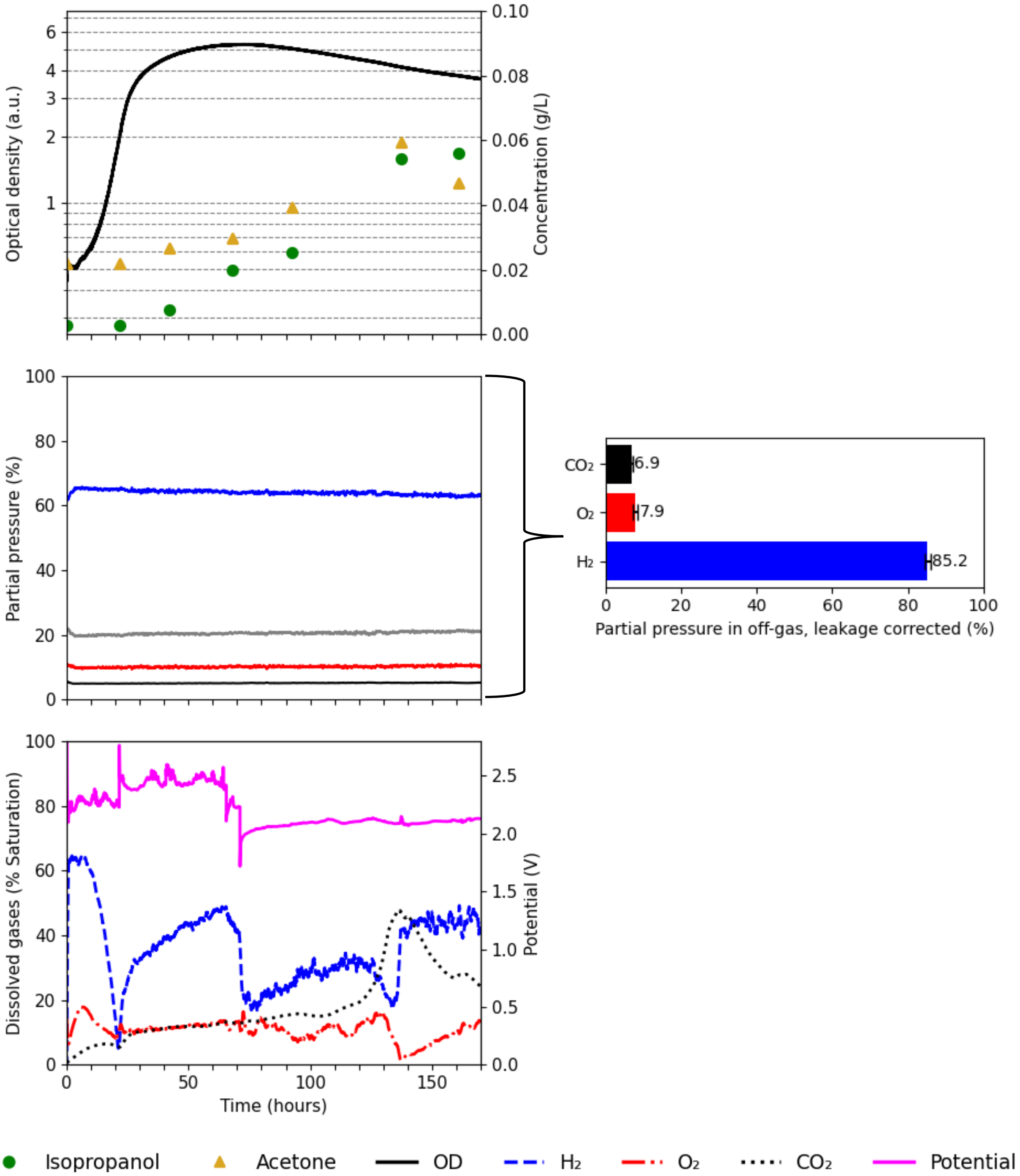

Figure S4. (related to Figure 2) Electricity driven cultivation of *Re*2133/pEG7c with adaptive current based on observed gas demand, with continuous off-gas data for demonstration of stability.

The culture was induced with 0.1 w/v % of arabinose at 24 hours. The current was adapted to the gas consumption of the culture, initially set to 15 mA cm^{-2}. During the growth phase between 21 and 66 hours, electrolysis was maintained at 30 mA cm^{-2} to mitigate H_2 or O_2 limitation. During the nitrogen depletion phase, between 80 and 170 hours, electrolysis was maintained at 5 mA cm^{-2} to improve efficiency and substrate consumption metrics, as the gas requirements were met at this lower current in absence of biomass formation. The presence of N_2 in the off-gas suggests air leakage, as N_2 is not actively added to the system. To account for this, the partial pressures of CO_2, O_2, and H_2 were recalculated. Since atmospheric air consists of approximately 78% N_2 and 21% O_2, the detected N_2 was used to estimate the corresponding O_2 introduced by the air leak. Both the N_2 and the proportional amount of O_2 were subtracted from the total gas composition, leaving only the contributions from CO_2, O_2, and H_2. The partial pressures of these gases were then normalized to reflect their corrected proportions in the absence of air leakage.

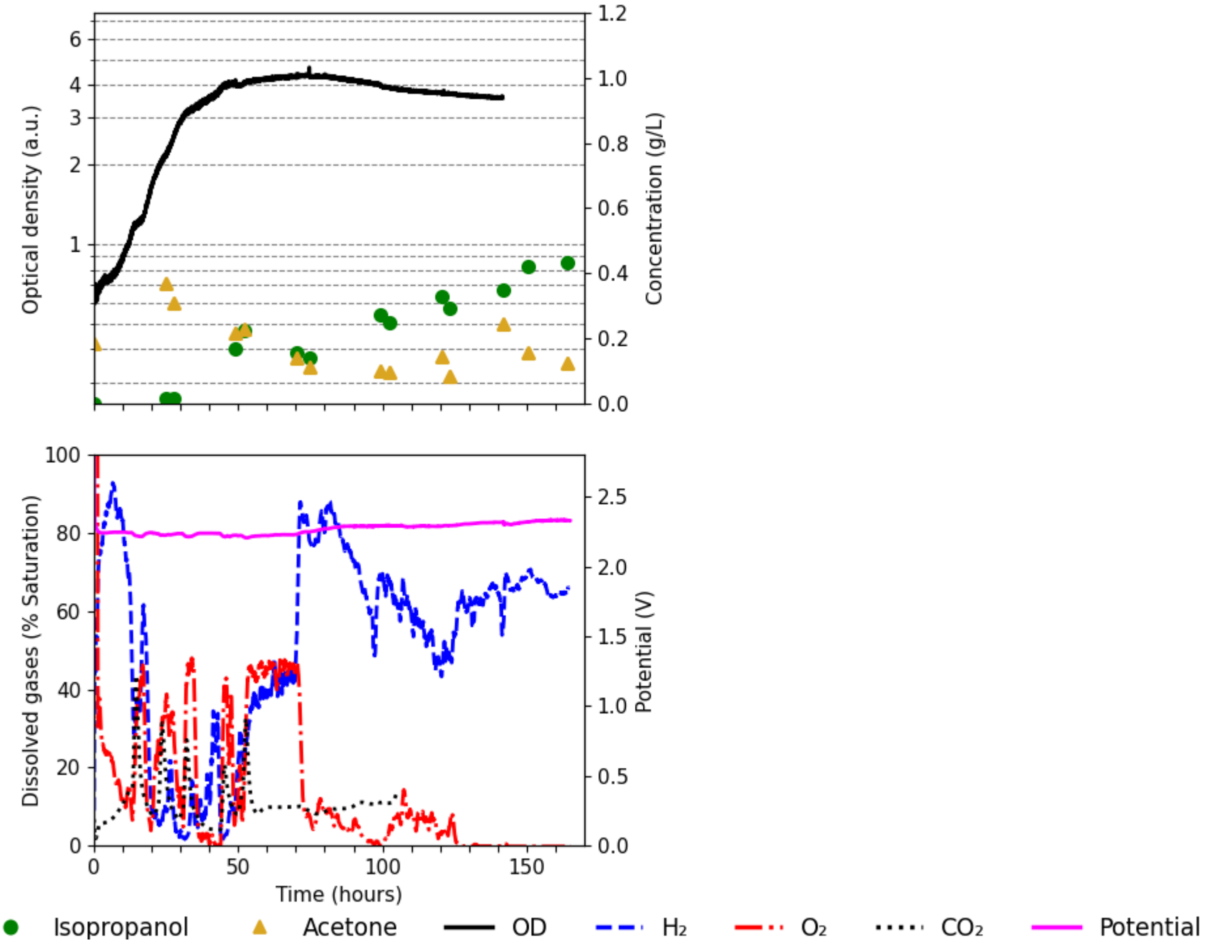

Figure S5. (related to Figure 2) Electro-driven cultivation of *Re*2133/pEG7c.
Electro-driven cultivation of *Re*2133/pEG7c, with adaptive O_2 supplementation based on observed gas demand between 0 and 70 hours.

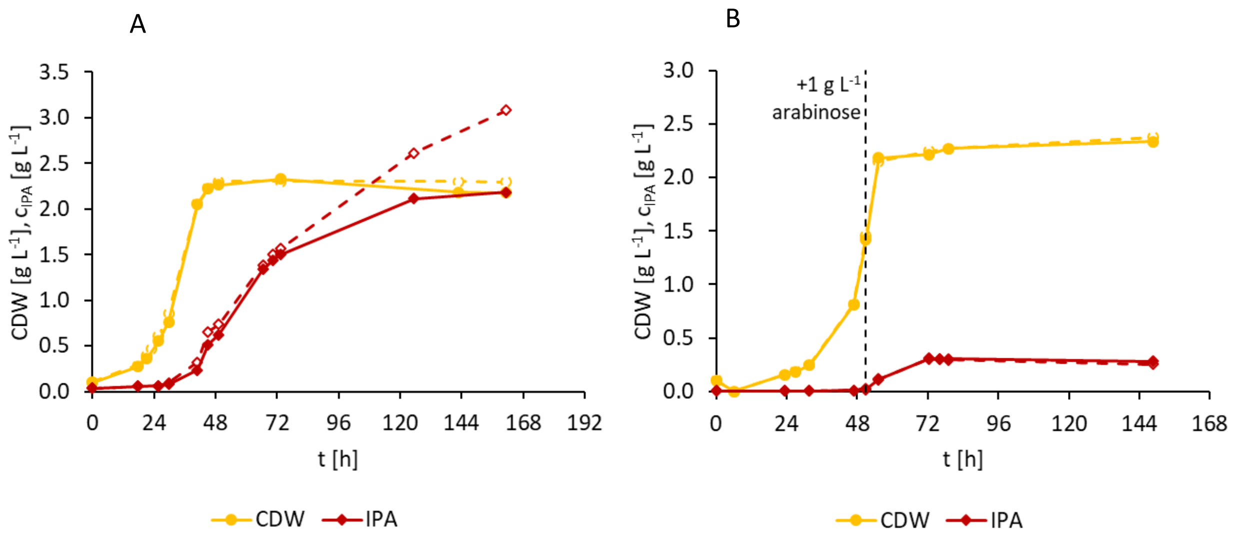

Figure S6. (related to STAR Methods: Isopropanol cultivation and sampling) Autotrophic batch cultivation showing growth and product formation of isopropanol-producing *C. necator* strains. Two *C. necator* strains were grown in autotrophic batch cultures, i.e. *Re*2133/pEG7b (A) and *Re*2133/pEG7c (B).

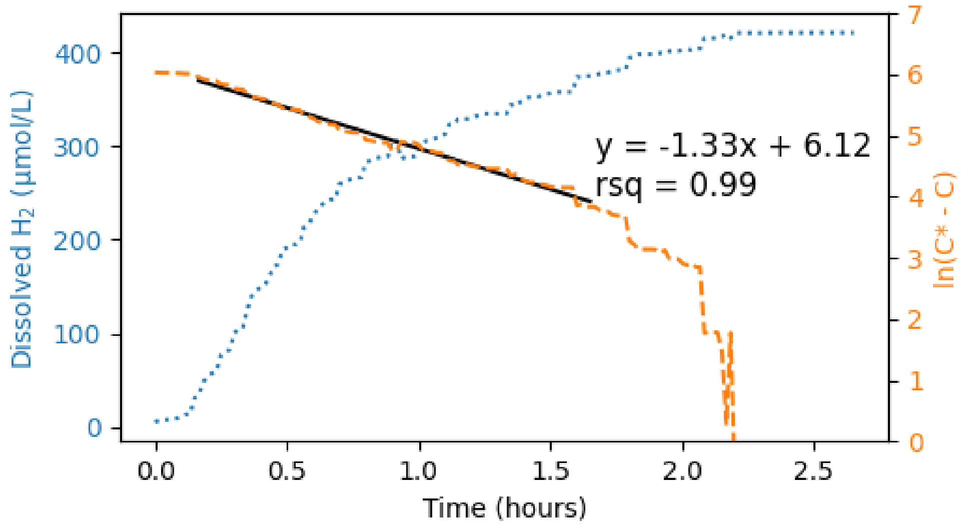

Figure S7. (related to STAR Methods: Determination of the Mass Transfer Constant) Determination of the mass transfer constant kLa for H_2.
The blue dotted line represents the dissolved H_2 concentration as it rises from 0 to equilibrium (C* = 420 µmol/L). The orange dashed line is the natural log transformed difference between C_{H2} and C_{H2}^*. The negative slope of the linear regression yields k_La = 1.33 h^{-1}.

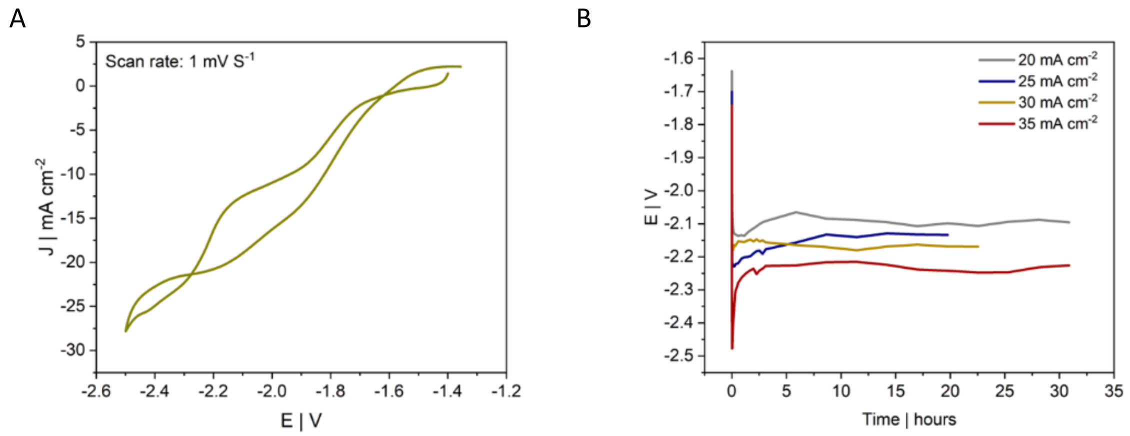

Figure S8. (related to STAR Methods: Electrochemical characterization) Abiotic PEM electrolyzer experiments.

A: Cyclic voltammetry of zero gap electrolyzer coupled to the bioreactor containing buffer as catholyte under abiotic control. B: Chronoamperometry at different current densities.

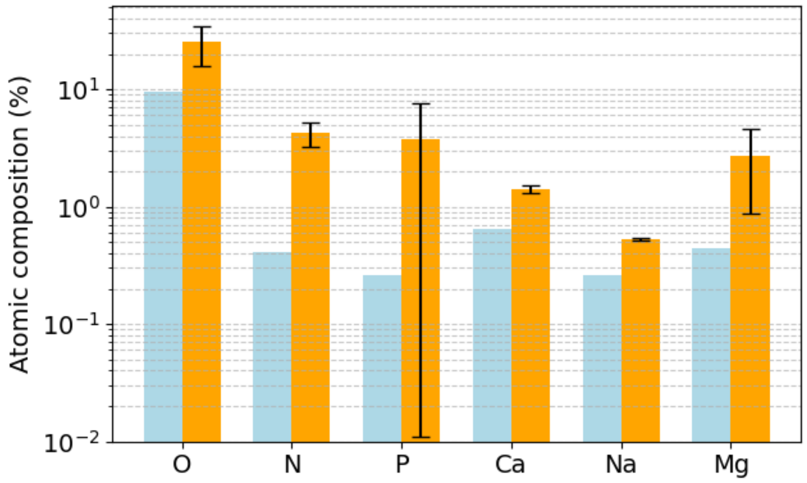

Figure S9. (related to Figure 3) Surface XPS of pentlandite cathode, fresh compared to post-mortem.

Blue bars (N=1) represent fresh electrodes, orange bars (N=3) represent post-mortem electrodes. Error bars represent the standard deviation.

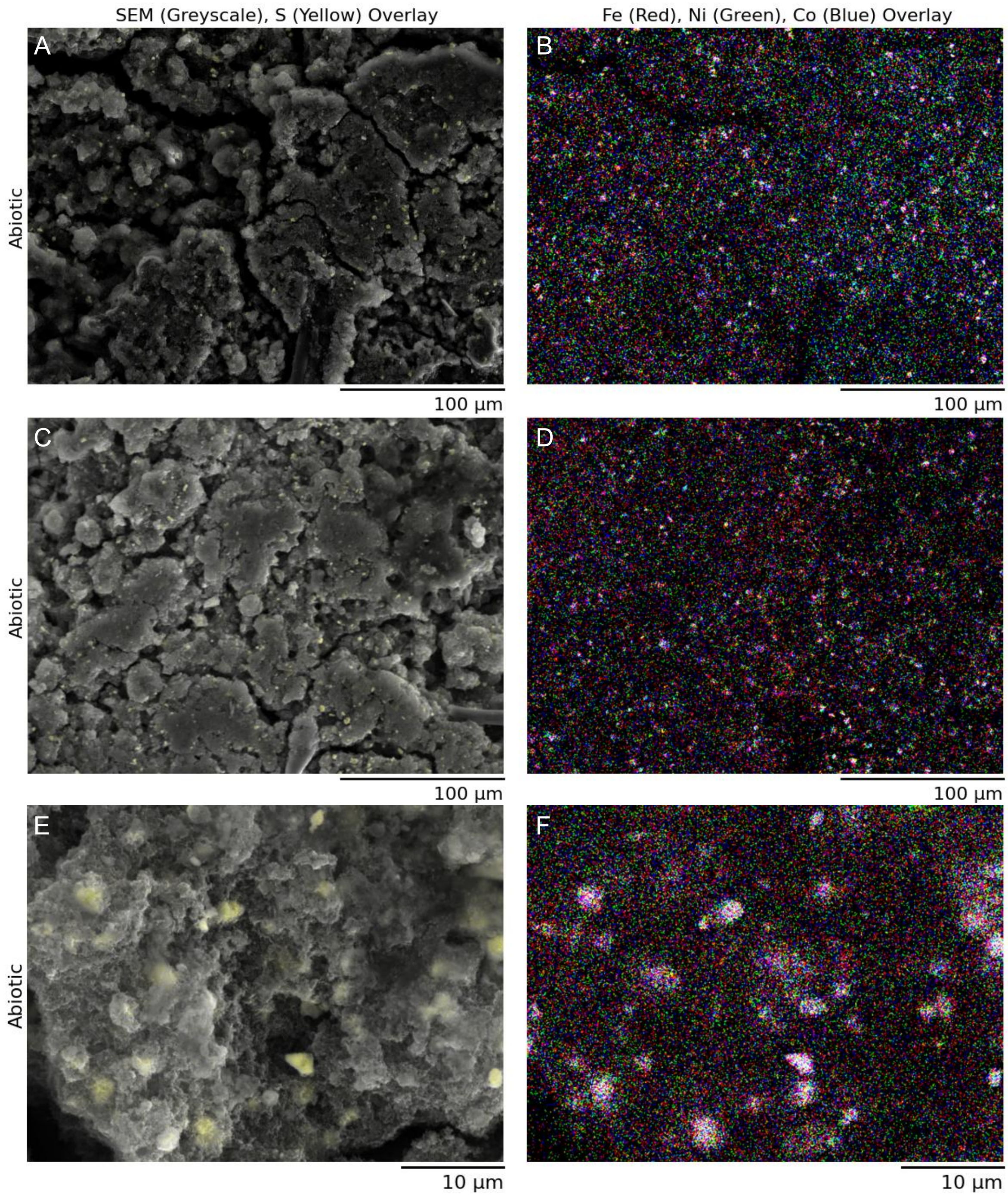

Figure S10. (related to Figure 4) Scanning Electron Microscopy (SEM) and Energy Dispersive X-ray (EDX) analysis of pentlandite ($Fe_3Ni_3Co_3S_8$) electrodes under abiotic and biotic conditions. Greyscale SEM images (A, C, E) are overlaid with EDX sulfur (yellow) to depict sulfur distribution on the electrode surface, while EDX maps (B, D, F) show the elemental distributions of iron (red), nickel (green), and cobalt (blue). The top row shows abiotic electrodes at a 100 µm scale, the middle row presents biotic electrodes at a 100 µm scale, and the bottom row shows biotic electrodes again, at a 10 µm scale to verify the absence of a biofilm.

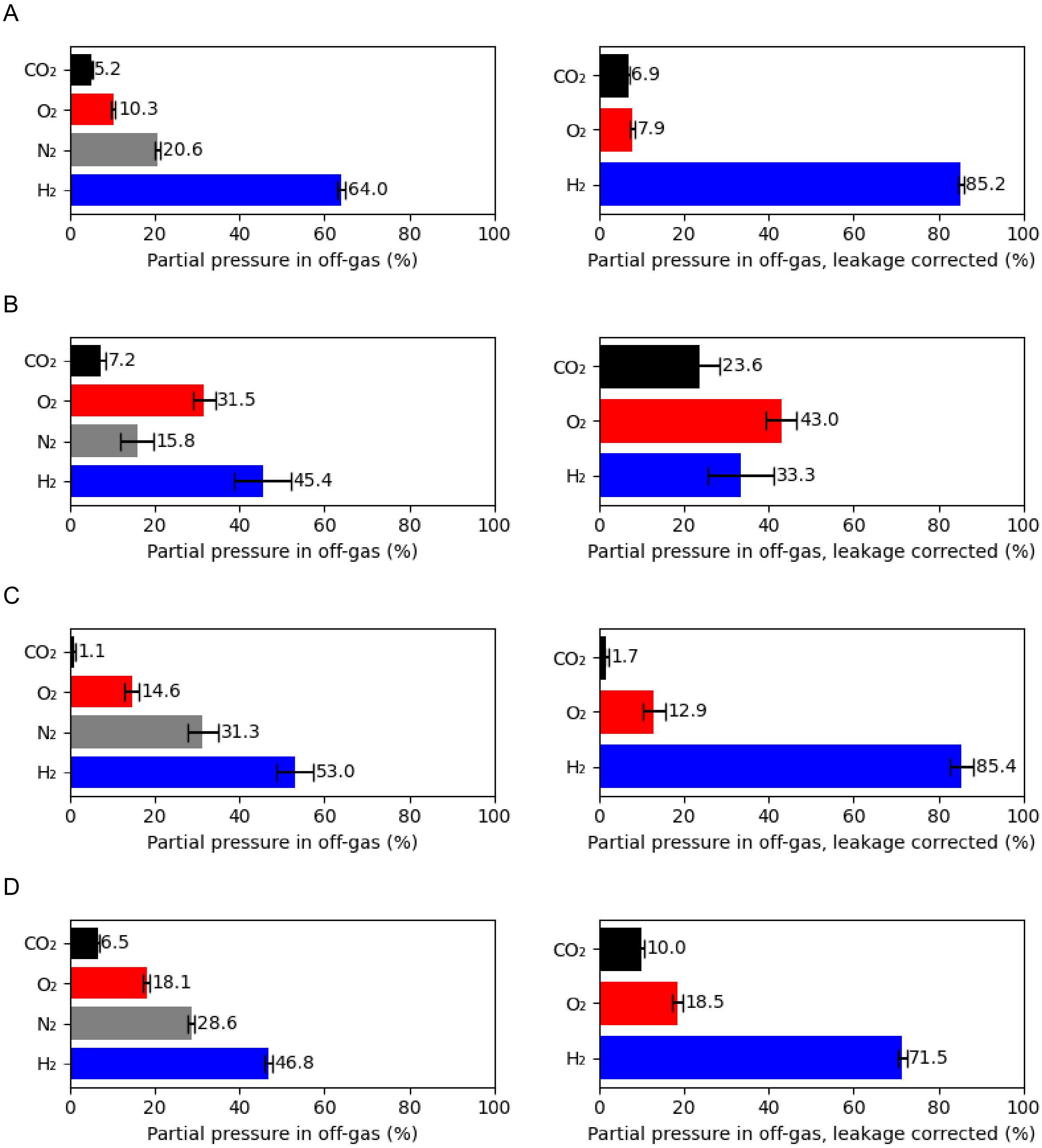

Figure S11. (related to Figure 2) Mean and standard deviations of the off-gas compositions for electro-driven cultivations.

The off-gas measurements corresponding to the cultivations shown in A) Figure S4, B) Figure S5, C) Figure 2A and Figure 2C, D) Figure 2B and 2D. Left) uncorrected and Right) corrected for air leakage reflected in the N_2 component. This data is related to “Analytical methods” in STAR Methods.

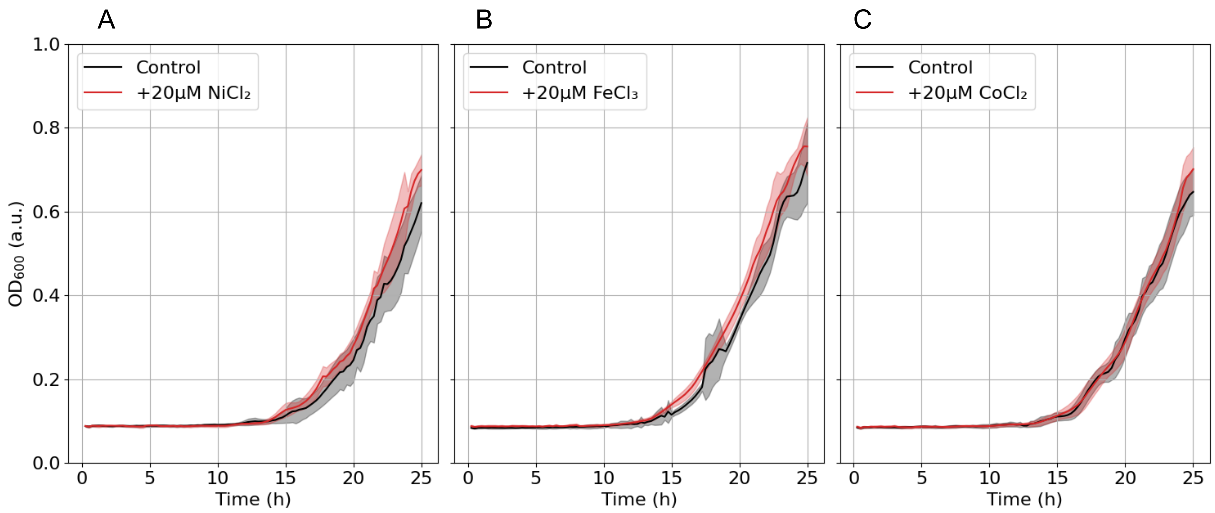

Figure S12. (related to STAR Methods: Determination of metal leaching toxicity) Determination of metal leaching toxicity.

Metal toxicity experiment of uninduced *Re*2133/pEG7c in MIT-medium supplemented with 200 µg/mL kanamycin, and A) 20 µM $NiCl_2$ B) 20 µM $FeCl_3$ C) 20 µM $CoCl_2$. Solid lines represent averages and shaded areas the standard deviations of 6 replicates. This data is related to “Determination of metal leaching toxicity” in STAR Methods.

Data S1. (related to Figure 2) Equations underlying gas transfer, electrochemical efficiency, and hydrogen energy calculations

$$PV = nRT$$

$$k_L a = -\frac{\Delta \ln(\mathrm{C}^* - \mathrm{C})}{\Delta \mathrm{t}}$$

$$\text{Faradaic Efficiency} = \frac{\mathrm{n} \cdot \mathrm{F} \cdot \mathrm{M}_{\text{product}}}{\mathrm{I} \cdot \mathrm{t}}$$

$$\text{moles of produced } H_2 = \frac{\eta \cdot I \cdot t}{n \cdot F}$$

$$E = n_{H_2} \times 286 \times 10^3 \text{ J}$$

where:

- *P*: Pressure (Pa or other appropriate units)
- *V*: Volume (L or other appropriate units)
- *n*: Number of moles
- *R*: Universal Gas Constant (8.314 J/(mol·K))
- *T*: Temperature (K)
- $k_L a$: Volumetric mass transfer coefficient (1/s)
- *C**: Saturation concentration of dissolved gas (mol/L or other appropriate units)
- C_L^{-}: Mean concentration of dissolved gas in the liquid phase (mol/L)
- *η*: Efficiency (dimensionless, given as 0.85)
- *I*: Electric current (A)
- *t*: Time (s)
- *F*: Faraday’s constant (96485 C/mol)
- M_{product}: Mass of the product formed (in grams).
